# Analysis of decolorization potential of *Myrothecium roridum* in the light of its secretome and toxicological studies

**DOI:** 10.1007/s11356-019-05324-6

**Published:** 2019-07-08

**Authors:** Anna Jasińska, Adrian Soboń, Aleksandra Góralczyk-Bińkowska, Jerzy Długoński

**Affiliations:** 1 Department of Industrial Microbiology and Biotechnology, Faculty of Biology and Environmental Protection, University of Lodz, Banacha 12/16, 90-237 Lodz, Poland; 2 Department of Microbial Genetics, Faculty of Biology and Environmental Protection, University of Lodz, Banacha 12/16, 90-237 Lodz, Poland

**Keywords:** Secretome, Laccase, Azo dyes, Decolorization, Detoxication, *Myrothecium roridum*

## Abstract

To identify the enzymes potentially useful for the decolorization of azo dyes, the secretome of the ascomycetous fungus *Myrothecium roridum* IM6482 was studied by using a bottom-up proteomic approach. Among the identified proteins, the most promising for dye removal was laccase, which decolorized respectively, 66, 91, 79, and 80% of Acid Blue 113 (AB 113), Acid Red 27 (AR 27), Direct Blue 14 (DB 14), and Acid Orange 7 (AO 7). The degradation of dyes was enhanced at the wide range of pH from 4 to 8. The addition of redox mediators allowed eliminating AB 113 in concentrations up to 400 mg/L and decolorization of the simulated textile effluent. Microbial toxicity and phytotoxicity tests indicated that dyes are converted into low-toxicity metabolites. This is the first insight into the *M*. *roridum* secretome, its identification and its application for removal of select azo dyes. Obtained results extended knowledge concerning biodegradative potential of ascomycetous, ligninolytic fungi and will contribute to the improvement of dye removal by fungi.

**Electronic supplementary material:**

The online version of this article (10.1007/s11356-019-05324-6) contains supplementary material, which is available to authorized users.

## Introduction

Azo dyes belong to the most commonly used group of dyes in the textile industry and constitute 60–70% of all dyestuff concerning textile production (Brüschweiler and Merlot 2017). Azo dyes have the general structure AR–N=N–R and often contain benzene, naphthalene, and aromatic heterocyclic and aliphatic rings. Some of these dyes are mutagenic and carcinogenic. According to the European Food Safety Authority, Direct Blue 14 (DB 14) commonly known as Trypan blue is possibly carcinogenic to humans (EFSA 2005). Acid blue 113 (AB 113) used as a dye in textile and leather products was proved to enter the human blood stream via the dermal route. It has been identified as a potential carcinogenic agent (Gupta et al. 2011). Another azo dye Acid Red 27 (AR 27) was widely used in cosmetic products, food coloring, fabrics, and leather. However, it has now been banned owing to its carcinogenic effects on humans. Moreover, recent studies have classified AR 27 as an endocrine disruptor.

Some dyes (application of which has been banned) are still present in the environment because of their accumulation in different areas or illegal use. However, even dyes with a non-toxic nature are not completely safe for humans. They can be transformed into toxic by-products by interactions with abiotic (hydrolysis, photolysis, and oxidation) and biotic (microbial activity) factors (Rawat et al. 2018). For example, a presumably non-toxic azo dye, Acid Orange 7 (AO 7), under saline conditions of the textile effluents, is converted into carcinogenic and/or mutagenic aromatic by-products such as aniline, 1-amino-2-naphthol, naphthalene, and phenyldiazene. The formation of genotoxic 5-amino-6-hydroxy-naphthalene-2-sulfonic acid and 4-amino-benzenesulfonic acid during the microbial degradation of the presumably safe Sunset Yellow dye has also been reported (Rawat et al. 2016). Thus, it is very important to eliminate these compounds from the environment.

Because of the environmentally friendly character and cost effectiveness, biodegradation driven by microorganisms is the most promising and intensively developing technology of pollutant removal. So far, the potential of bacterial azoreductases has been exploited in the decolorization and degradation of azo dyes (Mahmood et al. 2017). However, white-rot fungi belonging to Basidiomycetes and Ascomycetes and producing ligninolytic enzymes have also been proven to be able to degrade these toxic pollutants. The enzymatic treatment of dyes is very useful because of the action of the enzymes on dyes even when they are recalcitrant to the action of microorganisms. As most of the pollutants are hydrophobic compounds that cannot be absorbed for intracellular degradation, particularly useful are enzymes secreted extracellularly. In this context, the investigation of the fungal secretome, which consists of different enzymes with diverse functions, seems to be very important. Such a comprehensive approach is possible by using proteomic-based studies of the secretome. The identification of fungal proteomes allows us to find those proteins that can participate in the degradation process. For example, for the first time, proteomic study were applied to explain mechanisms of 4-n*-*nonylphenol (Szewczyk et al. 2014), alachlor (Szewczyk et al. 2015), and tributyltin (Soboń et al. 2016) biodegradation by fungi. A similar approach has been applied to the exploration of fungal proteomes to identify enzymes of biotechnological interest, examine the effects of heavy metals on the expression level of the secreted proteins, and investigate plant pathogenic fungi (Cherrad et al. 2012; Cologna et al. 2018; Pandey et al. 2018).

In the light of these considerations, for the first time, the investigation of the secretome of *M*. *roridum* in the search for enzymes involved in the decolorization of azo dyes was undertaken. The most likely candidate for this process turned out to be an enzyme with laccase activity, which was then successfully used to eliminate these toxic dyes from aqueous solutions. The enzyme had been previously characterized by us and preliminarily proven as potentially promising in the decolorization of Indigo Carmine (Jasińska et al. 2018). However, this study presents a holistic approach including the *M*. *roridum* secretome inspection to find dye-decolorizing enzymes, application of the laccase to azo dyes removal, and assessment of the toxicity of the metabolites formed after the treatment of the dyes.

## Materials and methods

### Chemicals

The azo dyes used in the decolorization studies were purchased from Sigma-Aldrich (USA). Their physicochemical characteristics are presented in Table S1. 2,2-Azinobis-3-ethylbenzothiazolin-6-sulfonic acid (ABTS); 2,6-dimethoxyphenol (DMP); 2,2,6,6-tetramethylpiperidine 1-oxyl (TEMPO); gallic acid (GA); caffeic acid (CA); and vanillin (V) were purchased from Sigma-Aldrich (USA). The other chemicals were obtained from Promega (USA), Serva (Germany), Bio-Rad (USA), and POCh (Gliwice, Poland). All reagents were analytical grade. Buffers and solutions were prepared in distilled water. For proteomic analysis, ultrapure water was used.

### Inoculum preparation and culture conduction

The filamentous fungus *Myrothecium roridum* IM 6482 was isolated from the soil, collected from around a textile dyeing factory, and stored in the strain collection of the Department of Industrial Microbiology and Biotechnology (University of Lodz, Poland) (Jasińska et al. 2012). Approximately 7 mL of the WHI3 medium was added to a fully sporulated culture slant. A spore suspension of approximately 5 × 10^9^ spores/mL was carried out at 28 °C with shaking at 140 rpm for 24 h. The precultures were transferred to the fresh medium (1:1 *v*/*v*), incubated for another 24 h, and used as an inoculum for enzyme biosynthesis. A modified Czapek–Dox medium (Jasińska et al. 2018) supplemented with 1 mM of copper sulfate or without the copper addition was used for fungus cultivation. The cultures were inoculated with 10% of a homogeneous preculture and performed for an appropriate period of time at 28 °C by shaking at 140 rpm. In the phase of the largest production of the enzyme, the fungal cultures were centrifuged. For the proteomic inspection of the secretome, proteins were precipitated by 20% trichloroacetic acid (TCA) according to the method described by Szewczyk et al. (2015). The proteins were dissolved in the standard sample solubilization buffer (SSSB), and the total protein content was measured using the Bradford method with bovine serum albumin (BSA) (Sigma, Germany) as the protein standard.

### Secretome separation in 2D electrophoresis

2D electrophoresis was conducted according to the procedure described by Szewczyk et al. (2015) with some modifications. The protein samples (600 μg) were loaded in 17-cm nonlinear IPG strips, pH 3–11 (Bio-Rad, USA). Isoelectric focusing (IEF) was performed using the Protean i12 IEF system (Bio-Rad, Germany) as follows: 50 V for 4 h, gradient to 6000 V for 5 h, and 50,000 Vh at 6000 V. The focused IPG strips were subjected to an additional reduction and alkylation treatment before the second dimension SDS-PAGE. The proteins were then separated in a 12% running gel by using a Protean XL cell (Bio-Rad, Germany) with the molecular mass marker of 6500–200,000 Da (Sigma-Aldrich, Germany) and stained using Coomassie blue. The gel analyses were performed using the Image Master 2D Platinum 7 software (GE Healthcare, Germany).

### In-gel tryptic digestion and protein identification by mass spectrometry

The in-gel tryptic digestion was performed according to the modified procedure described by Szewczyk et al. (2014). Protein spots were cut from the 2D gel, shredded, and placed into 1.5 mL protein low-bind tubes (Eppendorf, Germany). The gel pieces were decolorized using 50 mM ammonium bicarbonate in 50% acetonitrile (ACN) and dehydrated with ACN. The gel pieces were covered in the trypsin solution (Promega, Germany) and incubated overnight at 37 °C. The obtained peptides were extracted using 2% ACN with 0.1% trifluoroacetic acid (TFA) (15 min), 50% ACN with 0.1% TFA (60 min), and 90% ACN with 0.1% TFA (15 min). The extracts were combined, dried, and dissolved in 5 μL of 2% ACN with 0.1% TFA, and then, mixed with α-cyano-4-hydroxycinnamic acid. An AB Sciex 5800 TOF/TOF system (AB Sciex, USA) was used for the data acquisition. MASCOT and BLAST searches were performed according to Szewczyk et al. (2014). The proteins were identified against the NCBI database (restricted to the *Hypocreales* order, total number of sequences = 1,804,295) by using the Protein Pilot v4.5 software coupled with the MASCOT search engine v4.2.

### Decolorization of azo dyes

For the decolorization study, the secretome was precipitated overnight at 4 °C by using ammonium sulfate (80%). The proteins were dissolved in a 50-mM potassium phosphate buffer (pH 7.2), desalted, and concentrated by ultrafiltration with a 3-kDa cut-off (Ultra-15, Amicon, Bedford, MA, USA). Laccase was purified according to a method described previously (Jasińska et al. 2018). The decolorization experiments were performed in 96-well plates by using a reaction mixture containing the McIlvaine buffer (pH 2–8), laccase with activity toward ABTS (1 U/mL), various concentrations of the dye (50–400 mg/L), and mediators (100 μM). A decrease in the maximum absorbance of the dye at corresponding wavelength (Table S1) was monitored to determine its elimination. The decolorization percentage (DP) was calculated according to the following formula: DP(%) = (100 × (*A*_0_ − *A*_*t*_/A_0_)), where *A*_0_ and *A*_*t*_ are the initial absorbance of the reaction mixture and the absorbance after the incubation time, respectively.

### Toxicity tests

To evaluate the toxic effect of both the untreated and the treated dyes, a susceptibility toxicity assay was performed on the basis of the inhibitory growth of the reference bacterial strains of *Escherichia coli*, *Pseudomonas aeruginosa*, and *Staphylococcus aureus*. Each tested bacterial strain was first cultivated in the LB medium to obtain an optical density of a sample measured at a wavelength of 600 nm (OD_600)_ value of 0.1. Thereafter, the untreated and treated dye extracts in serial dilutions were separately added to the prepared bacterial broth and incubated at 37 °C. Changes in the OD_600_ value of each bacterial strain were recorded after 24 h of incubation. A negative control (the bacterial strain cultivated in the absence of the dye) was also designed for each experiment. The antimicrobial activities of dyes and their derivatives were expressed as a percentage of growth inhibition (GI%).

Phytotestkit (Tigret, Poland) was used to determine the direct effects of the untreated and treated dyes on the germination and early growth of *Sorghum saccharatum* and *Sinapis alba*. The assay was performed according to the protocol delivered by the producer. Briefly, seeds of the test plants were positioned on the test plate on the filter papers soaked with 20 mL of the water or treated or untreated dye solution. Plates were incubated at 25 °C (± 1 °C) for 72 h. Phytotoxicity experiments were conducted in three independent tests and each of them was performed using ten seeds of each plant. The germination percentage (GP) of the seeds and the growth of the roots and the shoots of the plant exposed on solutions of the untreated and treated dyes were assessed and compared with germination and growth in a control without chemicals.

### Statistical analysis

All experiments were conducted in three independent tests and each of them was performed in triplicate. Values are mean of all replicates. An average standard deviation (± SD) was calculated for each data. The *t* test using Excel 2013 (Microsoft Corporation, USA) was used to determine the significance of the differences between the samples.

## Results and discussion

### Secretome analysis for dye-decolorizing enzymes

**Fig. 1:**
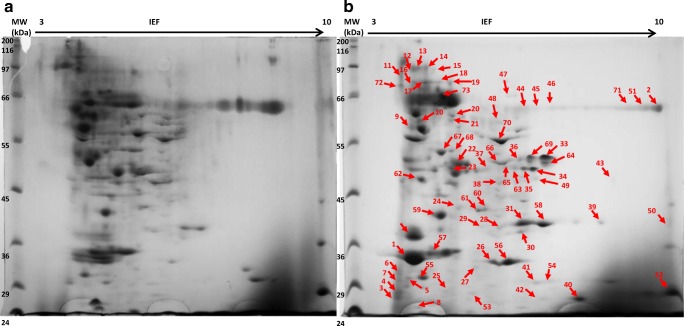
Representative 2DE maps of extracellular proteins isolated from **a** control and **b** copper-induced cultures of *M*. *roridum* cultivated in a modified Czapek–Dox medium for 48 h. Arrows denote protein spots expressed only under copper-induced conditions and identified using MALDI–TOF–MS analysis

To explore the *M*. *roridum* secretome and indicate the extracellular proteins potentially promising in the decolorization of azo dyes, a proteomic approach based on 2D electrophoresis (2DE) was applied. 2DE together with an MS analysis provides a powerful tool to separate, visualize, and identify hundreds of proteins at a time. The extracellular proteins of *M*. *roridum* from the culture cultivated with or without copper ions supplementation were extracted from 48-h cultures growing in the modified Czapek–Dox medium optimized for laccase production (Jasińska et al. 2018). The laccase activity was significantly increased by copper induction. The extraction of the extracellular proteome followed by 2DE separation and gel analysis using the ImageMaster 2D Platinum software revealed the presence of 336 and 266 proteins secreted by the fungus under non-induced and copper-induced conditions, respectively (Fig. 1). The distribution of the protein spots indicated that in both the cultures, most of the secreted proteins had an isoelectric point between 4 and 7 and a molecular mass of more than 29 kDa. The proteins induced by copper were identified and listed in Table 1. In general, in both cultures, the discovered proteins had a structural and regulatory function (e.g., ribosomal proteins, calmodulin, and Woronin body protein) and were involved in energy production and conversion processes (e.g., ATP synthase) as well as carbon metabolism (e.g., glucoside hydrolase, enolase, and glyceryl aldehyde-3-phosphate dehydrogenase). Also proteins involved in ligninocellulose degradation (such as glucoside hydrolase, alcohol oxidase, laccase, endoglucanases, and exocellulases), pathogenesis factors (e.g., calmodulin, glucan glucosidase, enolase, glyceryl aldehyde-3-phosphate dehydrogenase, and peptidyl-prolyl cis-trans isomerase), and proteins of the stress response (e.g., glutathione disulfide reductase, superoxide dismutase, heat-shock protein, aldo/keto reductase, and nucleoside diphosphate kinase) were identified. Beyond their housekeeping role in metabolism and cell development, many of the described enzymes have additional functions and are considered moonlighting proteins (Gancedo et al. 2016). Among the identified proteins, the most promising candidate for the degradation of azo dyes was laccase. Two laccase-like multicopper oxidases (LMCOs) produced by *M*. *roridum* IM 6482 were previously described by Jasińska et al. 2018. Their exclusive participation in the decolorization of Indigo Carmine was also proven by a new three-step approach involving an in-gel decolorizing potential screening with the use of Native PAGE, protein extraction, and molecular weight determination via SDS-PAGE. Laccase was also one of the factors involved in Malachite green biodegradation to non-toxic intermediates (Jasińska et al. 2012, 2015). A similar approach was proposed by Yu et al. (2015) in research on *Ganoderma lucidum*. Proteomic analyses of *G*. *lucidum* allowed to reveal that proteins which can play significant bioactive roles and provide a new foundation for the further functional investigations of this fungus. However so far, only a few reports have been published on the identification of fungal extracellular proteins in the context of their later application in biodegradation processes, including the decolorization of dyes (Cambri et al. 2016).

**Table 1:** Identification of the copper-induced proteins secreted by *M*. *roridum* during 48 h of cultivation in modified Czapek–Dox medium supplemented with 1 mM of copper sulfate. Proteins were identified using MALDI–TOF/TOF after 2D gel proteomic approach

Spot ID	Protein accession	MW (Da)	Mascot Score	Protein name	Species
1	KYK57333.1	37,745	50	Oxidoreductase family, NAD-binding Rossmann-fold protein	*Drechmeria coniospora*
2	KFY76980.1	80,590	45	Hypothetical protein V499_03500	*Pseudogymnoascus* sp. VKM F-103
3	EOO00854.1	18,363	146	Putative 40s ribosomal protein s11 protein	*Phaeoacremonium minimum* UCRPA7
4	EXL01076.1	14,819	211	40S ribosomal protein S22	*Fusarium oxysporum* f. sp. *raphani* 54,005
5	EWZ00769.1	16,986	208	Calmodulin	*Fusarium oxysporum* FOSC 3-a
6	KFA52822.1	15,913	190	Hypothetical protein S40293_00906	*Stachybotrys chartarum* IBT 40293
7	OAQ74584.1	11,805	77	60S ribosomal protein L30	*Purpureocillium lilacinum*
8	EFZ02691.2	14,136	226	Nuclear transport factor 2	*Metarhizium robertsii* ARSEF 23
9	CEJ79996.1	54,477	84	Putative glutathione disulfide reductase	*Torrubiella hemipterigena*
10	EQL02249.1	49,436	121	Glycoside hydrolase family 72 protein	*Ophiocordyceps sinensis* CO18
11	KDN61459.1	41,382	219	Putative glucan 1,3-beta-glucosidase, partial	*Colletotrichum sublineola*
12	Q5BF62.1	80,417	50	tRNA-dihydrouridine(47) synthase [NAD(P)(+)]	*Aspergillus nidulans* FGSC A4
13	OWY46879.1	66,349	61	Alcohol oxidase	*Alternaria alternata*
14	EEP82478.1	48,569	47	Conserved hypothetical protein	*Uncinocarpus reesii* 1704
15	CVL00965.1	77,835	72	Related to trehalase precursor	*Fusarium mangiferae*
16	CDP27663.1	38,808	110	Glycoside hydrolase family 131	*Podospora anserina* S mat+
17	GAP88032.1	81,635	69	Putative bifunctional catalase-peroxidase Cat2	*Rosellinia necatrix*
18	EGX95218.1	62,231	52	Laccase-1 precursor	*Cordyceps militaris* CM01
19	EGX95218.1	62,231	58	Laccase-1 precursor	*Cordyceps militaris* CM01
20	EFQ25737.1	56,686	64	Peptidase family M28	*Colletotrichum graminicola* M1.001
21	XP_007280335.1	50,845	67	Amine oxidase	*Colletotrichum gloeosporioides* Nara gc5
22	ETS01676.1	47,286	307	Putative enolase	*Trichoderma reesei* RUT C-30
23	KZZ98721.1	71,154	82	Heat-shock protein 70–2	*Aschersonia aleyrodis* RCEF 2490
24	KID61188.1	55,261	84	ATP synthase beta chain precursor, partial	*Metarhizium anisopliae* ARSEF 549
25	AIJ01039.1	29,080	70	Beta-tubulin 1, partial	*Trichoderma virens*
26	KND89895.1	15,768	217	Superoxide dismutase (Cu-Zn)	*Tolypocladium ophioglossoides* CBS 100239
27	O42724.4	15,879	53	Superoxide dismutase (Cu-Zn)	*Debaryomyces hansenii* CBS767
28	5GLX_A	22,727	63	Chain A, crystal structure of a glycoside hydrolase	*Thielavia terrestris* Nrrl 8126
29	EFY93910.1	27,186	185	Triosephosphate isomerase	*Metarhizium acridum* CQMa 102
30	KND91670.1	24,386	141	Superoxide dismutase (Mn)	*Tolypocladium ophioglossoides* CBS 100239
31	5GLX_A	22,727	78	Chain A, crystal structure of a glycoside hydrolase	*Thielavia terrestris* Nrrl 8126
32	KDB14292.1	24,645	303	Hypothetical protein UV8b_4887	*Ustilaginoidea virens*
33	ABQ42571.1	36,234	887	Glyceraldehyde-3-phosphate dehydrogenase	*Myrothecium gramineum*
34	Q92211.2	35,811	232	Glyceraldehyde-3-phosphate dehydrogenase	*Candida albicans* WO-1
35	Q9UVC0.2	36,187	111	Glyceraldehyde-3-phosphate dehydrogenase	*Wickerhamomyces ciferrii* NRRL Y-1031
36	ABQ42571.1	36,234	303	Glyceraldehyde-3-phosphate dehydrogenase	*Myrothecium gramineum*
37	EMR66584.1	37,423	59	Putative atp phosphoribosyltransferase protein	*Eutypalata* UCREL1
38	CCG25419.1	32,806	169	Gcy1 possible aldo/keto reductase	*Candida orthopsilosis*
39	EEU47353.1	21,737	203	Hypothetical protein NECHADRAFT_91822	*Nectria haematococca* mpVI 77-13-4
40	GAP82553.1	12,093	293	Putative peptidyl-prolyl cis-trans isomerase fkr-3	*Rosellinia necatrix*
41	EEU44844.1	16,912	192	Predicted protein	*Nectria haematococca* mpVI 77-13-4
42	OBR08967.1	12,153	154	Peptidyl-prolyl cis-trans isomerase	*Colletotrichum higginsianum* IMI 349063
43	EGY20812.1	172,583	60	Cortical actin cytoskeleton protein asp1	*Verticillium dahliae* VdLs.17
44	KGQ07345.1	52,876	94	Woronin body major protein	*Beauveria bassiana* D1-5
45	OOQ85253.1	52,790	49	Proline oxidase Put1	*Penicillium brasilianum*
46	GAD95962.1	27,092	50	Conserved hypothetical protein	*Byssochlamys spectabilis* No. 5
47	XP_016596054.1	74,298	62	Transketolase	*Penicillium expansum*
48	EWG42958.1	58,157	83	Hypothetical protein FVEG_04626	*Fusarium verticillioides* 7600
49	KLP04529.1	32,022	82	Putative dienelactone hydrolase family protein	*Fusarium fujikuroi*
50	KFH42761.1	49,928	112	Elongation factor 1-alpha-like protein	*Acremonium chrysogenum* ATCC 11550
51	XP_016600206.1	52,739	40	Proline oxidase	*Penicillium expansum*
52	OBR07004.1	26,741	100	EC5 protein	*Colletotrichum higginsianum* IMI 349063
53	KKP01860.1	15,060	121	Hydroxyisourate hydrolase	*Trichoderma harzianum*
54	EON98685.1	16,749	273	Putative nucleoside diphosphate kinase protein	*Phaeoacremonium minimum* UCRPA7
55	CCD33554.1	36,149	64	Similar to AHA1	*Botrytis cinerea* T4
56	KKY34952.1	15,834	473	Putative superoxide dismutase	*Diaporthe ampelina*
57	KLP04535.1	105,767	71	Putative heat-shock protein 70 (hsp70)	*Fusarium fujikuro*
58	5GLX_A	22,727	70	Chain A, crystal structure of a glycoside hydrolase	*Thielavia terrestris*Nrrl 8126
59	EFQ27121.1	19,059	86	Hypothetical protein GLRG_02292	*Colletotrichum graminicola* M1.001
60	KIE01117.1	55,267	183	ATP synthase beta chain precursor, partial	*Metarhizium majus* ARSEF 297
61	KKP00145.1	29,347	79	Hypothetical protein THAR02_07765	*Trichoderma harzianum*
62	OAQ78725.1	33,401	108	Phosphorylase family protein	*Purpureocillium lilacinum*
63	Q9UVC0.2	36,187	101	Glyceraldehyde-3-phosphate dehydrogenase	*Wickerhamomyces ciferrii* NRRL Y-1031
64	ABQ42571.1	36,234	356	Glyceraldehyde-3-phosphate dehydrogenase	*Myrothecium gramineum*
65	ORY58845.1	43,136	93	Vacuolar protease A	*Pseudomassariella vexata*
66	KND87524.1	61,800	229	Fructose-bisphosphate aldolase	*Tolypocladium ophioglossoides* CBS 100239
67	KFH48185.1	42,560	201	Vacuolar protease A-like protein	*Acremonium chrysogenum* ATCC 11550
68	ETS00085.1	54,841	408	ATP synthase F1, beta subunit	*Trichoderma reesei* RUT C-30
69	ABQ42571.1	36,234	728	Glyceraldehyde-3-phosphate dehydrogenase	*Myrothecium gramineum*
70	ETS87738.1	47,340	150	Enolase	*Pestalotiopsis fici* W106-1
71	ANH56452.1	62,931	76	Hypothetical protein, partial	*Hypocrella siamensis*
72	KDN61459.1	41,382	141	Putative glucan 1,3-beta-glucosidase, partial	*Colletotrichum sublineola*
73	EGE02315.1	30,008	44	Oxidoreductase	*Trichophyton equinum* CBS 127.97

### Decolorization of azo dyes in aqueous solutions

Laccases and LMCOs are used for the degradation of different toxic xenobiotic compounds (Rahmani et al. 2015; Daâssi et al. 2016; Legerská et al. 2018). Many reports have been published on the application of purified laccases or laccase-producing organisms to the elimination of synthetic dyes (Vats and Mishra 2017; Zhou et al. 2017; Bharagava et al. 2018). In this study, for the first time, laccase from *M*. *roridum* was used for the decolorization of industrially important azo dyes.

**Fig. 2:**
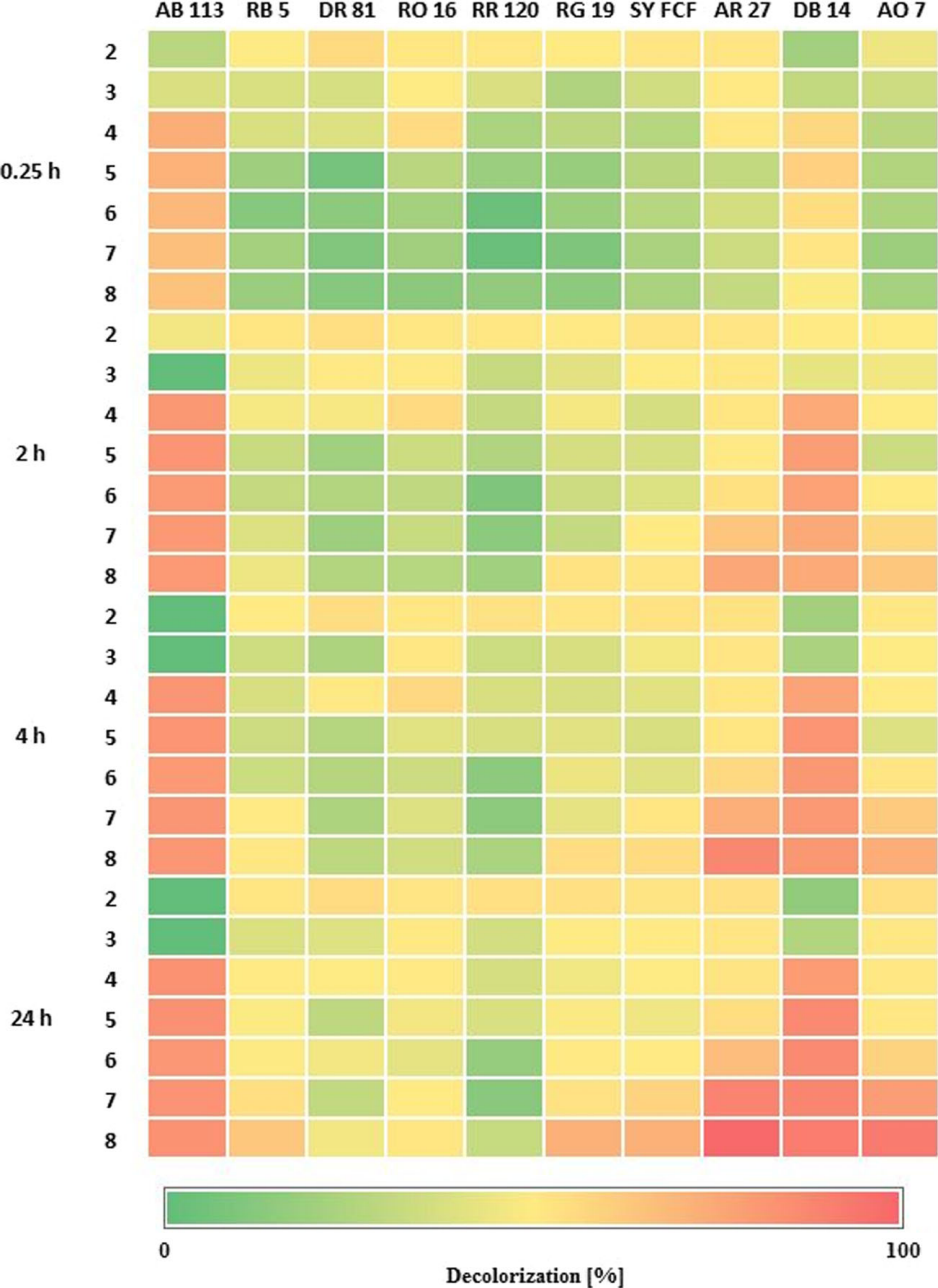
Heat map presentation of the averaged decolorization of azo dyes (50 mg/L) during 24-h incubation in McIlvaine buffer (pH 2–8) with 1 U/mL of *M*. *roridum* laccase

Both laccase-like enzyme activity and dye susceptibility to degradation depend on the pH value, thus the decolorization process was performed for 24 h in the aqueous solutions of the dyes with different pH values. Figure 2 shows a comparison of the decolorization efficiency obtained for particular dyes in the solutions with different pH values and at different time points. Values were given as a percentage and colored as a heat map to facilitate the comparison. The exact decolorization values are presented in Table S2. The *M*. *roridum* laccase decolorized all of the tested dyes. The dyes marked as DR 81 and RR 120 were the least susceptible to the decolorization, while the highest rates of removal were obtained for AB 113 and DB 14. After only 2 h of incubation in solutions with pH ranging from 3 to 8, the decolorization of both the dyes was more than 50–60%. Extending the incubation time to 24 h did not significantly affect the AB 113 decolorization, but allowed the decolorization of almost 80% of DB 14. Surprisingly, after 24 h of incubation, the decolorization of two different dyes AR 27 and AO 7 reached similar values. The enzymatic degradation of dyes was generally enhanced in the solutions with pH 4–8. Recently, laccase from *Myrothecium verrucaria* MD-R-16 demonstrated the decolorization of Methyl Red by more than 60% in the pH range of 4.5–6.5, with the maximum (80%) at pH 5.5 (Sun et al. 2017). As a general trend, most of the laccases of fungal origins maximally work at slightly acidic and neutral pH. For example, laccase from ascomycetous fungus *Paraconiothyrium variabile* decolorized azo dyes with extent higher than 50% in pH ranging from 4.5 to 5.5 (Mirzadeh et al. 2014). At pH 8, decolorization achieved only about 10%. The enzyme activity at higher pH is decreased because of the binding of the hydroxide anions to the T2/T3 coppers of laccase, thereby interrupting the internal electron transfer from the T1 to the T2/T3 centers (Baldrian 2006; Siroosi et al. 2016). However, the pH values of dye-containing effluents vary (even from the level of 3.9 to 14) depending on the dyeing step, effluent chemical composition, and the presence of dyeing auxiliaries (Dey and Islam 2015). Most biocatalysts cannot work under these conditions. Therefore, enzymes capable of catalyzing decolorization under these conditions are promising research objects.

### Decolorization of AB 113 in the presence of mediators

**Fig. 3:**
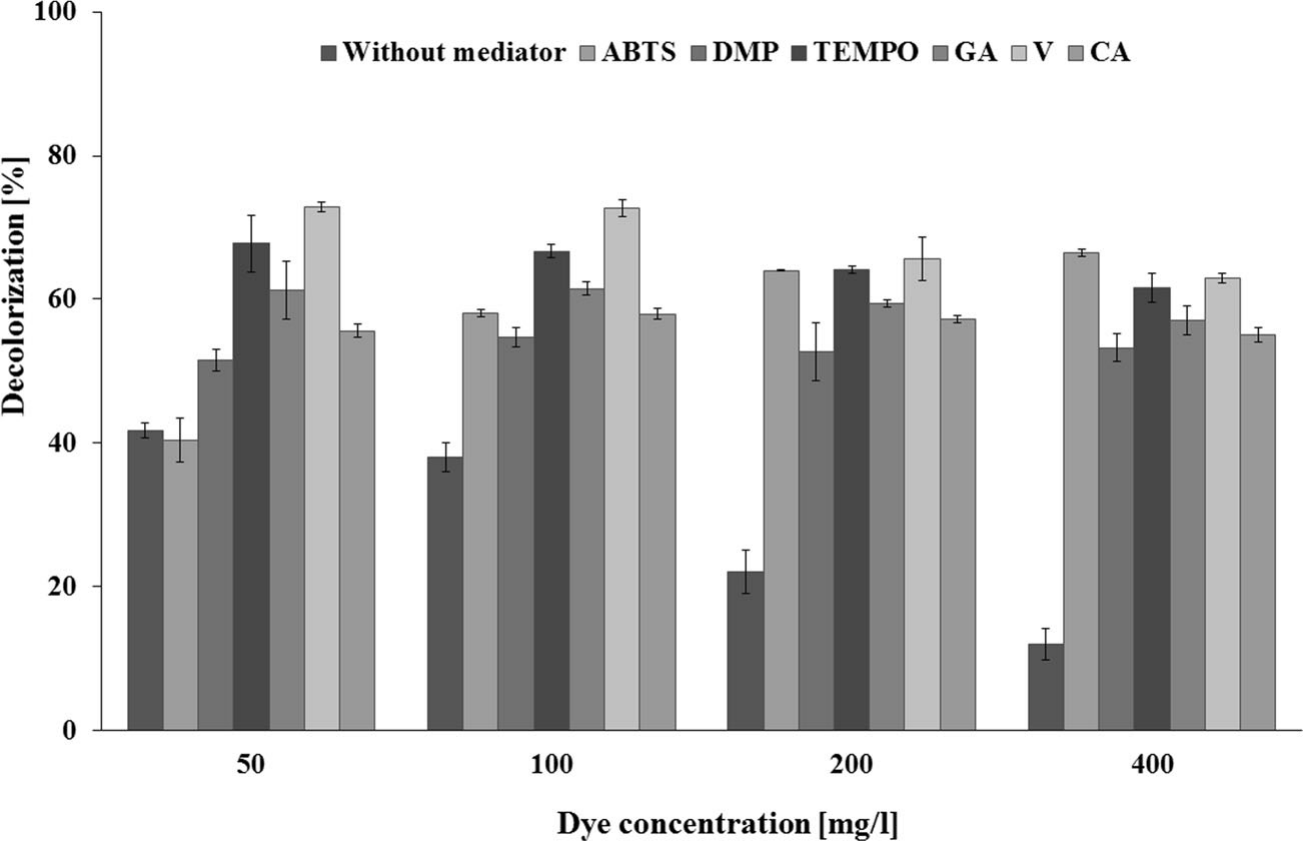
Effect of synthetic and natural redox mediators (100 μM) on AB 113 (concentration 50–400 mg/L) decolorization by *M*. *roridum* laccase (incubation time, 24 h; pH 8). Values are means ± standard deviation (SD). The significance of the differences between samples containing mediators and control samples was determined according to *t* test (*p* < 0.05)

Among all of the tested dyes, AB 113 was chosen as a model dye for further study. To accelerate the decolorization and remove higher concentrations of the dye, a reaction mixture containing laccase (1 U/mL) and AB 113 in the concentrations of 50, 100, 200, and 400 mg/L was supplemented with one of the synthetic (ABTS, DMP, or TEMPO) or natural (GA, V, or CA) mediators. Especially, mediators are useful for some azo and indigo dyes which are not suitable substrates for laccase-mediated degradation (Jasińska et al. 2016). As according to Fig. 3 all of the used mediators increased the AB 113 decolorization. The differences were mostly significant (*p* < 0.05) in solutions containing 400 mg/L of the dye. The addition of a mediator increased the decolorization by 4–5 times in comparison with the control samples (without a mediator). The highest decolorization of 400 mg/L AB113 was observed in the presence of ABTS and V (66% and 63%, respectively). However, because of its non-toxic character, V, a natural phenolic compound related to lignin polymers, was chosen for the further study. This is the first attempt to decolorize AB 113 with a concentration of as high as 400 mg/L in a laccase–mediator system. Until now mainly methods based on physicochemical processes have been used to eliminate it from contaminated wastewater (Rahmani et al. 2015; de Moura et al. 2016). AB 113 elimination in submerged fungal and bacterial cultures was described by Yang et al. (2016) and Nachiyar et al. (2012).

### Decolorization of dyes in the presence of additives and in stimulated textile effluent

Textile fabric manufacturing uses mixtures of dyes with various additives including solvents, antifoaming, whitening agents, and pH conditioners. Note that the addition of metals, detergents, chelating agents, or NaCl did not significantly decrease the AB 113 removal by the laccase of *M*. *roridum* (Table 2). Furthermore, a significant increase (by approximately 10–15%) in the level of decolorization was observed while comparing the samples containing surfactants such as SDS or Tween 80 against the control (*p* < 0.001). It suggests that the enzyme could be very useful in the surfactant industries. According to Akpinar and Ozturk-Urek (2017), surfactants can protect the enzyme structure and activity by forming micelles around the enzymes. The adverse effect of the non-ionic surfactant Merpol on the decolorization of Reactive Blue 19 by commercial *Trametes versicolor* laccase was observed (Champagne et al. 2010). Although the surfactant had no significant effect on the enzyme activity, the decolorization of Reactive Blue 19 was inhibited by increasing the surfactant concentration. In the present study, the decolorization of other dyes from the mixture containing an additive or from simulated textile effluent was also tested. These results showed that after 24 h of incubation of laccase with the simulated textile effluent, the total amount of AO 7, DB 14, AR 27, SY FCF, and AB 113 decreased by approximately 89%, 81%, 92%, 82%, and 89%, respectively. Further, the decolorization of DB 14, AO 7, AR 27, and SY FCF was not inhibited in the presence of additives. Commercial enzymes are often criticized for their limited efficiency and stability in the presence of laundry detergents. Thus, the application of enzymes effectively working in the surfactant-containing solution provides promising abilities. These results validated the applicability of the laccase-catalyzed degradation method for the treatment of real textile effluents.

**Table 2:** Decolorization of dyes (50 mg/L) after 24-h incubation *M*. *roridum* laccase (1 U/mL) with mixture containing single dye, dye with selected additives or with simulated textile effluent. Vanillin (concentration 100 μM) as redox mediator was used

Additive	Decolorization (%)									
	AB 113		DB 14		AO 7		SY FCF		AR 27	
Co^a^	71.92	± 1.43	89.65	± 0.22	82.20	± 0.39*	87.33	± 1.62*	83.97	± 2.14**
Cu^a^	71.85	± 1.82	87.23	± 5.46*	87.23	± 0.25*	85.13	± 2.28**	91.12	± 0.34
Zn^a^	71.59	± 1.48	89.84	± 1.60	87.60	± 0.69*	89.17	± 2.92	90.47	± 1.31
Cd^a^	72.21	± 0.92	90.24	± 0.74	87.88	± 0.33*	88.12	± 1.31	89.88	± 1.87
NaCl^a^	72.00	± 0.31	91.03	± 0.23	87.74	± 0.67	87.63	± 1.25	90.96	± 1.03
SDS^b^	89.23	± 0.08***	89.77	± 0.54	85.86	± 1.24	87.61	± 1.23*	88.41	± 2.80
EDTA^b^	72.87	± 0.53	90.63	± 0.42	87.78	± 1.81*	89.11	± 1.36	91.60	± 2.67
Uhrea^b^	73.43	± 2.43	90.77	± 0.67	86.41	± 0.63	83.47	± 1.43**	89.92	± 0.29
Tween 80^b^	85.94	± 0.38***	89.38	± 0.90	87.62	± 1.10*	89.15	± 0.27	90.86	± 0.92
Simulated textile effluent^c^	89.05	± 1.33***	90.98	± 1.67	88.61	± 1.12*	82.34	± 2.21***	91.51	± 0.98
Single dye	73.06	± 1.70	90.91	± 0.03	84.58	± 0.06	90.16	± 2.32	89.71	± 0.06

*AB 113*, Acid Blue 113; *DB 14*, Direct Blue 14; *AO 7*, Acid Orange 7; *SY FCF*, Sunset Yellow FCF; *AR 27*, Acid Red 27

Data are means ± SD. Significant differences (for mixtures containing additives relative to the single dye solution**)** are shown as asterisks (*p < 0.05; **p < 0.005, ***p < 0.001)

^a^0.1 M

^b^0.1%

^c^Containing all tested additives and azo dyes

### Toxicity study of AB113 and its intermediates formed during decolorization

The toxicity of AB 113 and its laccase-mediated decolorization products was evaluated by using a modified method with serial dilutions in the LB medium of the reference strains *E*. *coli*, *P*. *aeruginosa*, and *S*. *aureus*. The GI% of all of the concentrations of AB 113 was significantly reduced following the laccase treatment. The results of microbial toxicity are summarized in Table 3. Untreated AB 113 strongly inhibited the growth of all of the tested bacterial strains. The highest GI% was observed for *S*. *aureus*. In the LB medium containing 100 mg/L of the untreated dye, the growth of the bacteria was inhibited by 70%. In the medium containing the laccase-treated dye, GI was 14% lower and reached 56%. However, two Gram-negative bacteria *E*. *coli* and *P*. *aeruginosa* were found to be more sensitive to the dye and its intermediates. The GI of these strains assessed for 100 mg/L of AB 113 was 52% and 55%, respectively. In the presence of the dye intermediates, GI was approximately three times lower than that found for the parent dye. These findings suggested the reduction of the dye toxicity after laccase treatment. The antimicrobial activities of AB113 and the toxicity of its degradation products have not been assessed, thus far, with microbial toxicity assays. The toxicity of the stimulated textile effluent was established. The highest concentration of the simulated textile effluent extract completely inhibited the growth of all of the tested strains, while in the treated mixture, GI% was between 52 and 89%. This indicated the detoxication of the simulated textile effluent by *M*. *roridum* laccase. The decolorization of dyes did not always decrease their toxicity. For example, the toxicity of several untreated azo dyes (such as Direct Black 38, Direct Red 80, Reactive Black 5, Reactive Yellow 145, Acid Black 194, and Acid Red 266) was found to be lower than that of the laccase-treated intermediates (Mendes et al. 2011). However, from the environmental point of view, it is crucial that decolorization leads to the detoxication of the pollutant.

**Table 3:** Growth inhibition (%) of the reference bacteria cultivated for 24 h in LB medium containing serial dilutions of extracts of AB 113 or simulated textile effluent untreated (U) and treated with *M*. *roridum* laccase (T)

Dye	Concentration (mg/L)	Growth inhibition (%)					
		*E*. *coli*		*P*. *aeruginosa*		***S. aureus***	
		U^a^	T^b^	U^a^	T^b^	**U** ^a^	**T** ^**b**^
AB 113	6.25	9.33 ± 0.42	4.81 ± 0.55**	23.23 ± 1.62	7.07 ± 0.28**	43.84 ± 5.60	33.20 ± 5.83**
	12.50	11.70 ± 1.31	4.33 ± 0.27**	24.28 ± 1.28	9.80 ± 1.11**	38.97 ± 6.44	31.47 ± 5.22**
	25.00	13.46 ± 1.13	10.17 ± 1.37*	26.45 ± 0.11	12.54 ± 1.19*	44.04 ± 4.41	36.10 ± 1.64**
	50.00	29.76 ± 1.02	12.02 ± 0.68**	36.92 ± 2.53	13.18 ± 2.82**	66.49 ± 4.67	41.70 ± 2.73**
	100.00	52.58 ± 0.96	15.97 ± 1.79**	55.05 ± 2.88	20.04 ± 1.36**	70.52 ± 6.36	56.21 ± 0.00**
Simulated textile effluent	6.25	53.15 ± 7.91	20.78 ± 0.39**	59.17 ± 5.67	25.66 ± 2.42**	44.68 ± 1.51	34.46 ± 5.05**
	12.50	76.68 ± 0.30	66.47 ± 0.98**	76.29 ± 1.66	68.31 ± 1.21**	51.88 ± 1.97	37.83 ± 8.19**
	25.00	86.00 ± 1.98	56.78 ± 1.41**	84.52 ± 1.43	57.39 ± 0.90**	58.43 ± 2.64	39.37 ± 3.10**
	50.00	83.19 ± 3.99	60.75 ± 9.53**	81.15 ± 3.67	62.76 ± 7.25**	62.99 ± 3.92	48.45 ± 2.11**
	100.00	100.00 ± 0.00	87.18 ± 1.10**	100 ± 0.00	89.22 ± 1.74**	100 ± 0.00	52.92 ± 4.34**

Values are means ± standard deviation (SD). The significance of the differences between treated and untreated samples was determined according to *t* test (*p < 0.05; **p < 0.001)

^a^*U*, untreated dye

^b^T, laccase-treated dye

According to Rawat et al. (2016), most of the studies on microbial dye detoxification include assays with the involvement of one model organism only. However, a better solution seems to be the application of organisms belonging to different trophic levels (Rybczyńska-Tkaczyk et al. 2017). In the present study, the toxicity assessment was performed also with higher plants. The relative sensitivities of *S*. *saccharatum* and *S*. *alba* seeds toward AB 113 and the simulated textile effluent and their degradation products are summarized in Table 4. The results of the phytotoxicity study showed about 10% inhibition of the germination of the *S*. *saccharatum* and *S*. *alba* seeds soaked in the untreated dye, and respectively, 10 and 27% inhibition of the germination in the simulated textile effluent. The shoot lengths of plants germinated in the simulated textile effluent were found to be almost half lower than those of the plants germinated in the degradation metabolites. A significant increase in the lengths of the roots was observed in the extracted decolorization products (*p* < 0.05). These findings confirmed the reduced phytotoxicity of the textile effluent treated by the *M*. *roridum* laccase. Obtained results were well supported by the earlier findings (Verma et al. 2017; Chen et al. 2018) and indicated that the laccase-mediated dye degradation was an eco-friendly alternative to the conventional methods of dye degradation.

**Table 4:** Phytotoxicity of extracts of AB 113 or simulated textile effluent untreated (U) and treated with *M. roridum* laccase (T) along with water control

Dye		*S. saccharatum*			*S. alba*		
		GP[%]	Roots [cm]	Shoots [cm]	GP [%]	Roots [cm]	Shoots [cm]
Water		100.00±0.00	5.04±0.15	2.44±0.20	100.00±0.00	6.21±0.10	3.52±0.18
AB 113	U	93.33±5.77	3.93±0.21^ab^	1.67±0.42	93.33±5.77	5.54±0.41	2.98±0.20^ab^
	T	100.00±0.00	5.13±0.34^ab^	1.87±0.30	100.00±0.00	6.73±0.33	3.49±0.17^b^
Simulated textile effluent	U	90.00±10.00	1.70±0.45^ab^	0.80±0.10^ab^	73.33±5.77	3.05±0.13^a^	1.29±0.29^ab^
	T	100.00±0.00	3.72±0.68^ab^	1.31±0.20^ab^	100.00±0.00	5.00±0.11^a^	2.44±0.12^ab^

^a^Denotes a significant difference between water and tested solutions (p < 0.05)

^b^Denotes significant difference among untreated and laccase treated samples (p < 0.05)

## Conclusion

This is the first report exploring the secretome of *M*. *roridum* IM6482 and identifying enzymes with the potential decolorizing ability. According to obtained findings, the most promising for the azo dye decolorization was laccase. The degradation of dyes occurred in a wide range of pH. The decolorization considerably increased in the presence of redox mediators. Both single dyes and simulated textile effluent were successfully decolorized and detoxified. The obtained results extend the knowledge on fungal mechanisms of dye degradation and encourage further study on the scale-up of the bioprocess for the treatment of real textile effluents.

### Electronic supplementary material

ESM 1 (DOCX 69 kb)

## Appendix

E-supplementary data of this work can be found in online version of the paper.
