# Supplementary material for: Analysis of decolorization potential of Myrothecium roridum in the light of its secretome and toxicological studies
Source: Environ Sci Pollut Res Int. 2019 Jul 8;26(25):26313–23. doi: 10.1007/s11356-019-05324-6 (PMC6717178; doi:10.1007/s11356-019-05324-6)
Supplement: Supplementary file 1 — (DOCX 69 kb) [file 11356_2019_5324_MOESM1_ESM.docx]

**Analysis of decolorization potential of *Myrothecium roridum* in the light of its secretome and toxicological studies**

**Environmental Science and Pollution Research**

Anna Jasińska^1^*, Adrian Soboń^2^, Aleksandra Góralczyk-Bińkowska^1^, Jerzy Długoński^1^

^1^Department of Industrial Microbiology and Biotechnology, Faculty of Biology and Environmental Protection, University of Lodz, Banacha 12/16, 90-237 Lodz, Poland

^2^Department of Microbial Genetics, Faculty of Biology and Environmental Protection, University of Lodz, Banacha 12/16, 90-237 Lodz, Poland

*Corresponding author: e-mail: [anna.jasinska@biol.uni.lodz.pl](mailto:anna.jasinska@biol.uni.lodz.pl); Tel. +48426354717, Fax: +48426784932

**Table S1.** The physicochemical characteristics of azo dyes used in the presented study.

| **Abbreviation** | **C.I.Number** | **λ_max_ [nm]** | **Structure** |
| --- | --- | --- | --- |
| **AB 113** | Acid Blue 113 | 556 | [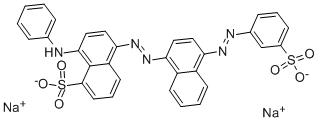](https://www.google.pl/url?sa=i&rct=j&q=&esrc=s&source=images&cd=&cad=rja&uact=8&ved=2ahUKEwiki-KWsczZAhWCFCwKHTM1DlwQjRx6BAgAEAY&url=http://www.chemicalbook.com/ChemicalProductProperty_EN_CB3250534.htm&psig=AOvVaw3Ab0HXQ34WvtJzG35cpqyq&ust=1520036979381301) |
| **RB 5** | Reactive Black 5 | 597 | 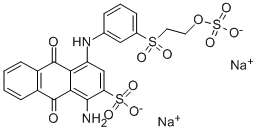 |
| **DR 81** | Direct Red 81 | 508 | 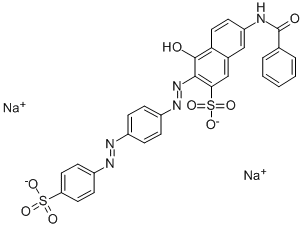 |
| **RO 16** | Reactive Orange 16 | 494 | 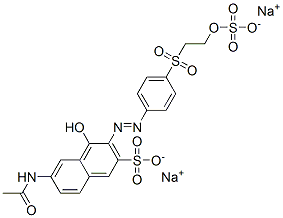 |
| **RR 120** | Reactive Red 120 | 535 | [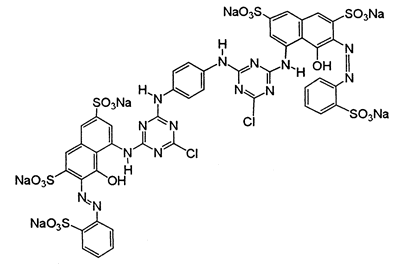](https://www.google.pl/url?sa=i&rct=j&q=&esrc=s&source=images&cd=&cad=rja&uact=8&ved=2ahUKEwiIobzfs8zZAhXIiSwKHdDZDyIQjRx6BAgAEAY&url=http://www.worlddyevariety.com/reactive-dyes/reactive-red-120.html&psig=AOvVaw3xV2zacmARZLR-NW_n5oOJ&ust=1520037665620905) |
| **RG 19** | Reactive Green 19 | 630 | [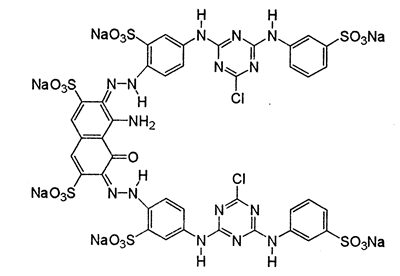](https://www.google.com/url?sa=i&source=images&cd=&cad=rja&uact=8&ved=2ahUKEwj5g6OgmYLbAhWDmLQKHa4bCwcQjRx6BAgBEAU&url=http://www.worlddyevariety.com/reactive-dyes/reactive-green-19.html&psig=AOvVaw0x2QpgG2dxeeLbflraujWz&ust=1526284030764440) |
| **SY FCF** | Sunset Yellow FCF | 482 | 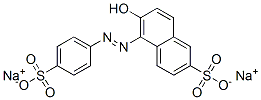 |
| **AR 27** | Acid Red 27 | 521 | 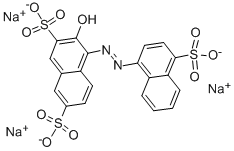 |
| **DB 14** | Direct Blue 114 | 607 | 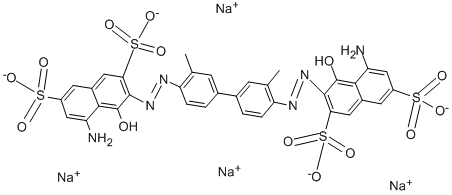 |
| **AO7** | Acid Orange 7 | 485 | 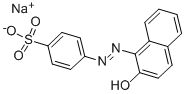 |

**Table S2.** Effect of pH and contact time on azo dyes decolorization by laccase from *Myrothecium roridum* IM 6482.

| **Time**  **[h]** | **pH** | **Decolorization [%]** | | | | | | | | | | |
| --- | --- | --- | --- | --- | --- | --- | --- | --- | --- | --- | --- | --- |
|  |  | **AB 113** | **RB 5** | **DR 81** | **RO 16** | **RR 120** | **RG 19** | **SY FCF** | **AR 27** | **DB 14** | **AO 7** |  |
| **0.25** | **2.2** | 6.65 | 12.28 | 22.66 | 13.79 | 14.23 | 12.69 | 16.04 | 16.05 | 4.85 | 10.64 |  |
|  | **3.0** | 9.08 | 8.95 | 8.74 | 11.92 | 9.04 | 5.76 | 8.34 | 13.11 | 7.26 | 8.14 |  |
|  | **4.0** | 49.32 | 8.96 | 9.37 | 21.05 | 5.58 | 6.76 | 6.35 | 14.38 | 24.04 | 6.53 |  |
|  | **5.0** | 47.11 | 4.61 | 1.43 | 6.60 | 4.27 | 4.14 | 6.54 | 7.21 | 28.53 | 6.01 |  |
|  | **6.0** | 43.11 | 2.73 | 3.32 | 5.07 | 0.73 | 4.45 | 6.34 | 8.53 | 19.93 | 5.66 |  |
|  | **7.0** | 38.22 | 5.04 | 2.29 | 4.71 | 0.54 | 2.12 | 5.42 | 8.14 | 15.89 | 4.41 |  |
|  | **8.0** | 36.43 | 4.19 | 2.68 | 3.12 | 3.57 | 3.22 | 5.49 | 7.43 | 11.67 | 5.14 |  |
| **2** | **2.2** | 10.89 | 15.17 | 19.31 | 14.86 | 14.15 | 13.09 | 16.23 | 15.85 | 12.70 | 12.11 |  |
|  | **3.0** | 9.00 | 10.53 | 13.40 | 13.63 | 7.59 | 9.65 | 11.96 | 14.71 | 10.01 | 10.97 |  |
|  | **4.0** | 63.00 | 11.15 | 11.22 | 22.65 | 7.47 | 11.12 | 8.64 | 15.12 | 51.52 | 12.24 |  |
|  | **5.0** | 65.08 | 7.71 | 4.74 | 7.94 | 5.87 | 8.69 | 8.73 | 13.44 | 60.25 | 8.10 |  |
|  | **6.0** | 61.03 | 7.43 | 6.14 | 7.11 | 2.14 | 8.14 | 9.23 | 18.47 | 56.65 | 12.89 |  |
|  | **7.0** | 62.50 | 9.32 | 4.54 | 7.66 | 3.17 | 7.49 | 12.51 | 35.16 | 52.91 | 24.51 |  |
|  | **8.0** | 61.98 | 10.60 | 6.18 | 6.31 | 4.76 | 17.23 | 15.78 | 53.98 | 51.60 | 34.15 |  |
| **4** | **2.2** | 10.20 | 13.01 | 20.50 | 14.73 | 17.88 | 16.06 | 16.99 | 16.95 | 4.97 | 14.59 |  |
|  | **3.0** | 8.90 | 8.26 | 5.73 | 14.00 | 8.01 | 8.85 | 11.05 | 15.57 | 5.46 | 11.91 |  |
|  | **4.0** | 64.36 | 8.82 | 13.19 | 23.00 | 8.80 | 8.92 | 9.61 | 15.49 | 55.72 | 12.50 |  |
|  | **5.0** | 65.08 | 8.06 | 6.22 | 9.56 | 8.89 | 9.71 | 8.81 | 15.43 | 64.70 | 9.25 |  |
|  | **6.0** | 61.66 | 8.03 | 6.26 | 8.09 | 3.26 | 10.47 | 9.44 | 23.39 | 63.26 | 15.95 |  |
|  | **7.0** | 64.07 | 12.83 | 5.70 | 9.36 | 3.33 | 9.96 | 15.20 | 49.18 | 62.22 | 32.26 |  |
|  | **8.0** | 63.76 | 14.62 | 6.95 | 8.30 | 5.56 | 20.90 | 22.11 | 72.72 | 63.22 | 50.24 |  |
| **24** | **2.2** | 10.38 | 15.19 | 21.53 | 15.79 | 19.04 | 18.12 | 16.54 | 18.09 | 3.67 | 19.28 |  |
|  | **3.0** | 8.83 | 9.10 | 9.47 | 13.39 | 8.44 | 11.98 | 12.99 | 15.87 | 6.16 | 14.22 |  |
|  | **4.0** | 66.09 | 12.65 | 12.12 | 13.58 | 8.65 | 10.84 | 11.88 | 16.38 | 60.83 | 14.38 |  |
|  | **5.0** | 67.39 | 11.60 | 6.98 | 10.99 | 8.97 | 11.59 | 10.74 | 20.18 | 70.81 | 13.87 |  |
|  | **6.0** | 63.68 | 11.76 | 10.91 | 9.88 | 3.87 | 13.45 | 12.70 | 40.47 | 71.22 | 26.90 |  |
|  | **7.0** | 65.95 | 19.41 | 7.31 | 12.57 | 3.02 | 17.60 | 26.36 | 75.19 | 75.00 | 59.71 |  |
|  | **8.0** | 66.25 | 34.01 | 10.92 | 15.44 | 7.57 | 48.01 | 47.79 | 91.56 | 79.53 | 80.91 |  |
